# Tracking Different States of Spiked Environmental DNA Using Multiplex Digital PCR Assays

**DOI:** 10.1111/1462-2920.70086

**Published:** 2025-03-28

**Authors:** Julia Zöhrer, Judith Ascher‐Jenull, Andreas O. Wagner

**Affiliations:** ^1^ Department of Microbiology Universität Innsbruck Innsbruck Austria; ^2^ Department of Experimental Architecture, Integrative Design Extremes Universität Innsbruck Innsbruck Austria

**Keywords:** *Bacillus subtilis*, *Escherichia coli*, extracellular DNA, intracellular DNA, spike‐and‐recovery controls

## Abstract

The study of microbial communities based on the total environmental DNA (eDNA) is influenced by the presence of different eDNA states, i.e., intracellular (iDNA) and extracellular DNA (exDNA), and the choice of the DNA extraction method. Although the use of spike‐and‐recovery controls facilitates the diagnosis of such issues, appropriate experimental setups simultaneously accounting for the different eDNA states and their bacterial origins are missing. Here, we used two single‐gene deletion mutants of both *Escherichia coli* and *Bacillus subtilis* to trace exDNA and iDNA spike‐ins of each selected model organism within environmental samples. Unique primer/probe sets were developed for each strain, allowing their absolute quantification using multiplex digital PCR assays. The proposed spike‐and‐recovery controls were successfully applied to various environments including soil, sediment, sludge and compost. While the percent recovery of spiked iDNA differed significantly between *E. coli* and *B. subtilis* , results were similar for both model organisms in the case of spiked exDNA, emphasising that the fate of DNA molecules in the environment is similar irrespective of their bacterial origin. Hence, future studies may benefit from the proposed approach to better understand methodological ambiguities related to the eDNA extraction in general as well as the separation of the different eDNA states.

## Graphical abstract

Spike‐and‐recovery controls for the extraction of environmental DNA simultaneously accounting for different DNA states and bacterial origins were developed. The approach was successfully applied to various environmental samples. Overall, the percent recovery of intracellular DNA spike‐ins differed among species, while results were similar in terms of spiked extracellular DNA.

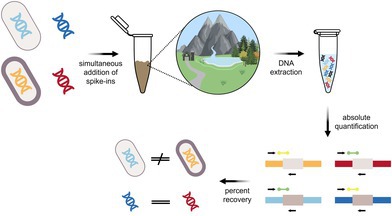

## 1 Introduction

The introduction of molecular methods (e.g., PCR, next‐generation sequencing) in environmental sciences has revolutionised the field of microbial ecology, gaining insights into the (meta)genomes of uncultivable organisms and whole biomes. In fact, the extraction of DNA from environmental samples (i.e., eDNA) and subsequent downstream analyses enable investigations on both the presence and functional potential of single species, populations or even complex microbial communities within different habitats (Carini et al. 2016; Emerson et al. 2017; Pawlowski et al. 2020; Taberlet et al. 2012; Torti et al. 2015; Wagner et al. 2015). However, different states of eDNA exist: intracellular DNA (iDNA) and extracellular DNA (exDNA). While the former is located within structurally intact cells and therefore potentially living cells, the latter is present outside thereof in the extracellular environment. Hence, exDNA originates from iDNA either by active secretion from living organisms or passive release mechanisms induced by cell lysis after cell death (Levy‐Booth et al. 2007; Nagler et al. 2020; Nagler, Insam, et al. 2018; Pietramellara et al. 2009; Torti et al. 2015). Once released into the extracellular environment, exDNA may persist also for relevant periods of time, ranging from a few hours up to several months and even thousands of years (Agnelli et al. 2007; Dell'Anno and Corinaldesi 2004; Levy‐Booth et al. 2007; Nielsen et al. 2007; Pietramellara et al. 2009). The persistence is tightly linked to the binding of exDNA molecules onto organic/inorganic particles and the concomitant protection against enzymatic degradation (Agnelli et al. 2004; Alawi et al. 2014; Corinaldesi et al. 2005; Levy‐Booth et al. 2007; Nielsen et al. 2007; Ogram et al. 1988, 1994; Pietramellara et al. 2009; Probst et al. 2021; Torti et al. 2015). For example, the fate of exDNA has been repeatedly investigated in soils and it was found that both the properties of the DNA molecules itself (e.g., length, conformation, purity) and soil characteristics such as mineralogy, water content, temperature and ionic composition determine its persistence (Levy‐Booth et al. 2007; Ogram et al. 1988, 1994; Pietramellara et al. 1997, 2001, 2007, 2009; Saeki et al. 2010). In this context, previous studies reported that up to 90%, 80% and 60% of the total eDNA pool in deep‐sea sediments, surface water and forest soils occur as exDNA, respectively (Agnelli et al. 2004; Dell'Anno and Danovaro 2005; Lennon et al. 2018). However, this aspect has often been neglected in studies based on the direct extraction and subsequent downstream analyses of the total eDNA (Probst et al. 2021; Wagner et al. 2015).

Moreover, analyses based on the total eDNA pool may not only be inflated by the presence of different eDNA states, but also by the choice of the DNA extraction method, which determines the reliability of obtained results (Albertsen et al. 2015; Geraldi et al. 2020; Roopnarain et al. 2017; Starke et al. 2019; Wagner et al. 2015). Given the high diversity of prokaryotic and eukaryotic microorganisms in environmental samples, this has been frequently observed when comparing the strength and rigidity of bacterial cell walls. Compared to gram‐negative bacteria, gram‐positive species possess a thicker cell wall, which is characterised by multiple crosslinked peptidoglycan layers, and therefore, they seem to be less accessible during DNA extraction (Albertsen et al. 2015; Robe et al. 2003; Roopnarain et al. 2017; Starke et al. 2019). Even though not yet considered as standard procedure for DNA‐based analyses, the use of spike‐and‐recovery controls facilitates the diagnosis of species‐specific issues related to DNA extraction efficiency (Stoeckel et al. 2009). In fact, the selection and implementation of appropriate spike‐and‐recovery controls have proven challenging as they must not occur naturally in the selected environmental samples and behave similarly to the focal organisms during DNA extraction. For example, organisms harbouring plasmid‐borne expression or antibiotic resistance genes are commonly used as spike‐in controls (e.g., McKinney and Dungan 2020; Stoeckel et al. 2009; van Frankenhuyzen et al. 2013). Given the high microbial diversity, however, the use of a single organism may not satisfy the requirements (Harrison et al. 2021; Stoeckel et al. 2009). In this context, Stämmler et al. (2016) deployed a set of extremophilic bacteria as spike‐ins to study the murine gut microbiome as they are naturally not present therein under physiological conditions. Besides being not present in the environment, another major limitation of cellular spike‐ins is that the selected organisms must be easily cultivated and quantified. Especially the absolute quantification of related target genes may be inflated due to the occurrence of copy number variations which are difficult to control (Harrison et al. 2021; Tourlousse et al. 2017; Zemb et al. 2020). Similarly, targeting plasmid‐encoded genes hampers consistent and accurate quantification as the number of plasmids may vary considerably between cells and therefore allows only a rough estimation (Jahn et al. 2016; McKinney and Dungan 2020; Stoeckel et al. 2009). Likewise, a series of genetically modified microbes containing an artificial DNA sequence in their genomes has been developed as internal sample process controls for the detection and quantification of pathogens by quantitative PCR, avoiding false‐negative results (e.g., Chen et al. 2023; Kobayashi et al. 2013; Rossmanith et al. 2011; Murphy et al. 2007). Moreover, chimeric DNA sequences have been introduced as spike‐and‐recovery controls, mainly in the context of absolute microbiome profiling (e.g., Hardwick et al. 2018; Tkacz et al. 2018; Tourlousse et al. 2017; Zemb et al. 2020). Despite the ability to design and alter these synthetic DNA sequences arbitrarily, they solely reflect the recovery of DNA fragments without accounting for the efficiency of cell lysis during DNA extraction (Harrison et al. 2021; Tourlousse et al. 2017; Zemb et al. 2020).

Hence, the ultimate aim of this study was to establish spike‐and‐recovery controls for the extraction of DNA from environmental samples which account for both (i) the different states of eDNA (i.e., iDNA vs. exDNA) as well as (ii) the bacterial amenability to cell lysis in the case of spiked iDNA (i.e., gram‐negative vs. gram‐positive bacteria). Indeed, microbial communities are highly diverse and not only made up of bacteria but also other pro‐ and eukaryotic microorganisms. However, the present study will focus on bacterial organisms as they allow the precise quantification of spike‐ins without inducing any methodological biases related to their growth and/or lifecycle. In comparison, many fungal and other eukaryotic microorganisms show a multicellular/filamentous growth, have a dimorphic lifecycle, or any form of sexual reproduction in the sense of changing their ploidy, hampering their reliable use as spike‐ins. Furthermore, the differentiation between gram‐negative and gram‐positive organisms enables in‐depth analyses of species‐specific differences within a single domain of life, being representative of a broad set of microorganisms. For this purpose, two individual strains were selected from single‐gene deletion mutant libraries for each of the model organisms *Escherichia coli* and *Bacillus subtilis* . While one strain was used as a cellular spike‐in (i.e., iDNA), the other was subjected to genomic DNA (gDNA) extraction prior to its use as spike‐in (i.e., exDNA). Even though the strains possess the same antibiotic resistance cassette, they can be distinguished by quantitative PCR methods such as digital PCR (dPCR), specifically targeting the terminal ends of the resistance cassette and adjacent flanking regions as these boundaries are unique to each strain. Concomitantly, this allows the simultaneous tracking of both model organisms and states using multiplex dPCR assays. Besides evaluating the applicability of the designed spike‐and‐recovery controls to randomly selected representative environmental samples for molecular microbial biodiversity monitoring, we aimed to assess (i) the minimum number of added spike‐ins, which is necessary to reliably track the respective target genes using a commercial DNA extraction kit and (ii) the percent recovery of spiked exDNA in relation to its fragment length.

## 2 Material and Methods

### 2.1 Reference Strains

**FIGURE 1:**
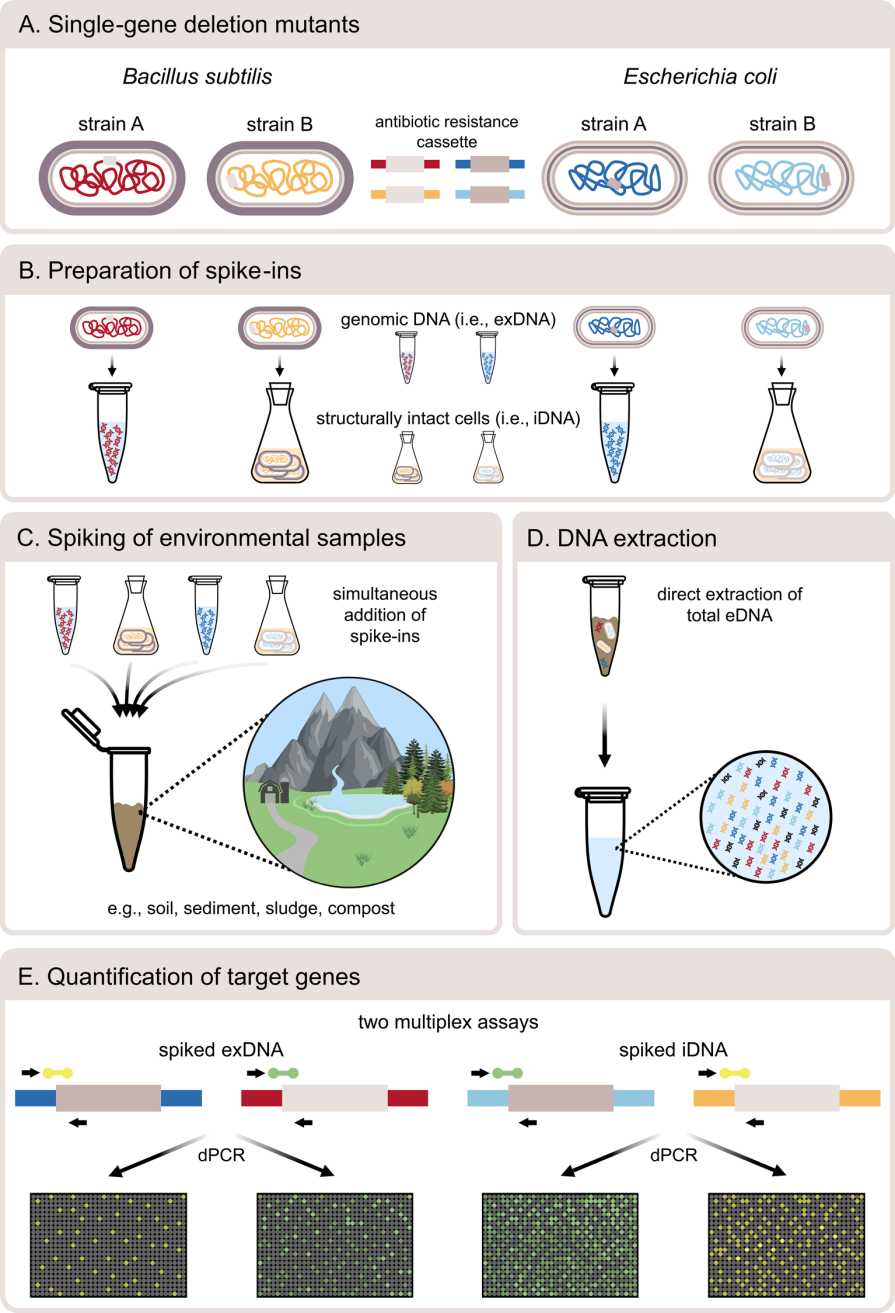
Workflow of the proposed spike‐and‐recovery experiments. (A) Two strains of both *E. coli* and *B. subtilis* were selected from single‐gene deletion mutant libraries. (B) For both model organisms, one strain was used as cellular spike‐in (i.e., intracellular DNA = iDNA), whereas the other was subjected to genomic DNA extraction prior to its use as spike‐in (i.e., extracellular DNA = exDNA). (C) The different states of spike‐ins were added simultaneously at predefined concentrations to different environmental samples, including soil, sediment, sludge and compost. (D) To test the applicability of the proposed method, the environmental DNA (eDNA) of spiked samples was extracted directly using a commercial DNA extraction kit. (E) Two multiplex digital PCR (dPCR) assays were developed to specifically quantify the spiked target genes. Whereas one assay was designed to simultaneously track iDNA spike‐ins the other quantified spiked exDNA. For each target gene, forward and reverse primers were selected to bind within the antibiotic resistance cassette and the adjacent 5′ or 3′ flanking region, respectively, while the hydrolysis probe was chosen to cover both sites.

Two well‐studied model organisms, *E. coli* and *B. subtilis* , were used as spike‐and‐recovery controls. More precisely, the selection of two genetically modified strains for each organism allowed the simultaneous tracking of both exDNA and iDNA spike‐ins within a single experiment while considering their gram‐negative and gram‐positive origin. Therefore, suitable strains of *E. coli* K‐12 BW25113 and *B. subtilis* subsp. *subtilis* str. 168 were chosen from single‐gene deletion mutant libraries (Baba et al. 2006; Koo et al. 2017), which were obtained from Horizon Discovery Ltd. (Waterbeach, United Kingdom) and the Bacillus Genetic Stock Center (Columbus, United States), respectively. For *E. coli* , the strains *∆bglA::kan* (JW2869, 6‐phospho‐β‐glucosidase A) and *∆chbF::kan* (JW1723, 6‐phospho‐β‐glucosidase) were selected to represent spiked exDNA and iDNA, respectively. Similarly, the mutants *∆licH::kan* (BKK38560, 6‐phospho‐β‐glucosidase) and *∆bglA::kan* (BKK40110, aryl‐6‐phospho‐β‐glucosidase) were chosen as exDNA and iDNA spike‐ins of *B. subtilis* . These strains are characterised by deletions of single, non‐essential genes which were replaced by a kanamycin resistance cassette. Even though this resistance cassette is similar for the selected strains of each model organism, its genomic location and hence its terminal ends and adjacent 5′ and 3′ flanking regions are unique, allowing specific differentiation (Figure 1A).

### 2.2 Preparation of eDNA Spike‐Ins

The target strains of *E. coli* and *B. subtilis* were grown overnight in Lysogeny Broth (LB) medium (1% tryptone, 0.5% yeast extract, 0.5% NaCl) supplemented with 7.5 and 30 μg mL^−1^ kanamycin, respectively (Baba et al. 2006; Koo et al. 2017).

#### 2.2.1 Extracellular DNA Spike‐Ins

The exDNA spike‐ins were artificially generated by extracting the genomic DNA (gDNA) from pure bacterial cell cultures, using the NucleoSpin Soil Kit (Macherey‐Nagel, Düren, Germany) according to the manufacturer's recommendations (Figure 1B). Prior to DNA extraction, 1.5 mL of the pure culture broth was centrifuged at 5000 × g for 2 min to pellet the cells and reduce the sample volume. After removing the supernatant, cell disruption was performed using a FastPrep‐24 5G Instrument (MP Biomedicals, Santa Ana, United States) together with Lysis Buffer SL2 and 100 μL Enhancer SX. Quality and quantity of extracted gDNA were determined by UV/VIS spectrophotometry (NanoDrop 2000c, Thermo Scientific, Waltham, United States). Based on the amount of extracted gDNA and the genomic size of the selected *E. coli* (4,631 kbp) and *B. subtilis* (4,215 kbp) strains, the number of genome equivalents (μL^−1^) and thus single‐copy target genes were calculated.

To evaluate the recovery of spiked exDNA depending on its fragment length, solely gDNA extracts of the selected *B. subtilis* strain were considered. Encompassing the target region for subsequent digital PCR (dPCR) assays, primer sets were designed to amplify 127, 1197 and 12,821 bp DNA fragments (Table S1). PCR was conducted using the Q5 High‐Fidelity DNA Polymerase (New England Biolabs, Ipswich, United States). Reaction setup and thermocycling conditions were chosen according to the manufacturer's recommendations. After checking the correct size of the PCR products by agarose gel electrophoresis (1%, 100 V, 25 min), purification was performed using the HiYield Gel/PCR DNA Fragment Extraction Kit (Süd‐Laborbedarf GmbH, Gauting, Germany) according to the manufacturer's recommendations. Quality, quantity and copy number of purified PCR products were determined as described for extracted gDNA.

#### 2.2.2 Intracellular DNA Spike‐Ins

Strains representing iDNA were used as cellular spike‐ins (Figure 1B). Hence, overnight bacterial cultures were centrifuged at 1000 × g for 5 min to harvest cells. The supernatant was discarded, and the remaining cell pellet was washed with 0.9% NaCl solution to remove both membrane‐compromised cells as well as any dissolved DNA as a source of exDNA. The resuspended cells were again centrifuged at 1000 × g for 5 min, and the supernatant was discarded as before. The removal of dissolved DNA in the supernatant of washed cells was checked fluorometrically using a Quantus Fluorometer (Promega, Madison, United States) in combination with the QuantiFluor dsDNA System (Promega, Madison, United States). Cell suspensions were considered free of dissolved DNA if the measured DNA concentrations were lower than or equal to blank. However, as centrifugation at 1000 × g does not pellet all cells, the supernatant was filtered through sterile, regenerated cellulose membrane filters (0.2 μm, Phenomenex, Torrance, Unites States) to avoid interference with DNA measurement. Related biases caused by the removal of dissolved DNA during filtration were tested separately and therefore excluded (*data not shown*). As a result, three subsequent cycles of cell washing and centrifugation were considered appropriate to remove any dissolved DNA, which was analysed in triplicate for both *E. coli* and *B. subtilis* . Subsequently, the washed cells were resuspended in 5 mL 0.9% NaCl, and cell viability was assessed by live‐dead staining using the LIVE/DEAD *Bac*Light Bacterial Viability Kit (L7007, Thermo Fisher Scientific, Waltham, United States) according to the manufacturer's recommendations. Fluorescence images of three independent cell suspensions were analysed for each model organism, and the proportion of viable cells was calculated, which was similar for both *E. coli* (95.9% ± 2.27%) and *B. subtilis* (96.2% ± 1.92%). Finally, the cell number (μL^−1^) of washed cell suspensions was determined by using a Thoma counting chamber (Assistant, Glaswarenfabrik Karl Hecht, GmbH & Co KG, Sondheim, Germany).

### 2.3 Spiking Different eDNA States

#### 2.3.1 Environmental Samples

The applicability of the proposed spike‐and‐recovery controls was tested using a total of four randomly selected environmental samples, including soil, sediment, sludge from anaerobic digestion and compost, covering some of the most representative ecosystems for microbial biodiversity monitoring. Aquatic environments were deliberately omitted in this context as DNA extraction methods differ compared to the selected environmental samples, hampering the direct comparability of obtained results. All sampling sites were located in Tyrol, Austria. Soil and sediment samples were collected from an agricultural meadow (47°7′21.2″ N, 10°37′51.9″ E) and the river Inn (47°15′34.7″ N, 11°19′39.5″ E), respectively. Using a corer device (Ø = 5 cm), 5 subsamples were taken at each site at a sampling depth of 10–20 cm. Subsequently, subsamples were pooled to create a composite sample for each site. Both compost and sludge samples derived from a green‐ and biowaste treatment plant (47°13′48.1″ N, 10°49′51.1″ E). Likewise, 5 subsamples were taken from a compost pile and pooled to create a composite sample, while the sludge sample was collected directly at the outlet of a full‐scale digestion plant, as described previously (Prem et al. 2021). All samples were immediately transported to the laboratory, where they were sieved at 2 mm. Aliquots of each environmental sample were stored at −20°C prior to the addition of spike‐ins.

#### 2.3.2 Experimental Procedure for eDNA Spiking

After determining the number of cells and genome equivalents within washed cell suspensions and DNA extracts of *E. coli* and *B. subtilis* , respectively, the spike‐ins were diluted at desired concentrations and added to 200 mg of the selected soil, sediment, sludge and compost samples (Figure 1C). To test the minimum number of added spike‐ins that is necessary to reliably track the respective target genes, concentrations of 10^5^, 10^6^, 10^7^ and 10^8^ cells and genome equivalents of both *E. coli* and *B. subtilis* were used. However, instead of adding exDNA and iDNA spike‐ins for each model organism separately, a homogeneous mixture containing the exDNA spike‐ins of both *E. coli* and *B. subtilis* was prepared. Similarly, iDNA spike‐ins were mixed. Finally, 25 μL of each mixture was added to the four selected environmental samples. In addition, the effect of DNA fragment length on the recovery of spiked exDNA was tested—based on pilot experiments—on soil and sludge samples, by adding 3 × 10^7^ copies of target genes within a volume of 25 μL.

After adding the spike‐ins, samples were inverted carefully five times and subsequently incubated at room temperature for 15 min, allowing adsorption of spiked exDNA and iDNA to the environmental matrices (McKinney and Dungan 2020; Thomson‐Laing et al. 2022). All experiments were performed in triplicate.

#### 2.3.3 Extraction of Total eDNA

The total eDNA was directly extracted from spiked environmental samples using the NucleoSpin Soil Kit (Macherey‐Nagel, Düren, Germany). This DNA extraction kit was supposed to be well suited for a wide range of environments, as better results were achieved within different environments compared to other DNA extraction kits (e.g., Desneux and Pourcher 2014; Knauth et al. 2013; Roopnarain et al. 2017; Wagner et al. 2015). However, minor modifications of the manufacturer's recommendations were performed: Cell disruption was conducted using a FastPrep‐24 5G Instrument (MP Biomedicals, Santa Ana, United States) in combination with Lysis Buffer SL1 and 75 μL Enhancer SX (Figure 1D), as shown previously to result in high recovery rates (Wagner et al. 2015). The quality and quantity of extracted eDNA were determined by UV/VIS spectrophotometry (NanoDrop 2000c, Thermo Scientific, Waltham, United States).

### 2.4 Quantification of Target Genes

#### 2.4.1 Development of Target‐Specific Primers and Probes

Prior to the design of specific primer/probe sets for each of the four target genes, reference sequences of wildtype strains *E. coli* K‐12 BW25113 (CP009273.1) and *B. subtilis* subsp. *subtilis* str. 168 (NC_000964.3) were obtained from GenBank (Sayers et al. 2024). Sequences of deleted genes were replaced by the respective kanamycin resistance cassette for each of the spiked eDNA states using Benchling (https://www.benchling.com/). Following sequence amplification and verification of the kanamycin resistance cassette and adjacent flanking regions (Table S2), primers and probes were developed for the absolute quantification of each strain using Primer3Plus (Untergasser et al. 2012). Therefore, forward and reverse primers were selected to bind within the kanamycin resistance cassette and the 5′ or 3′ adjacent flanking regions, while the probe was chosen to cover both sites. Instead of designing four independent assays for each of the spiked eDNA states and bacterial organisms, we intended to develop two multiplex assays with two parallel reactions each, one for spiked exDNA and the other for spiked iDNA (Figure 1E, Table 1). Self‐ and heterodimer formation between any primer and probe combinations was examined using Benchling. Additionally, the Primer‐BLAST tool (Ye et al. 2012) was used to test the specificity of the designed assays in silico.

**TABLE 1:** Primer and probe sequences designed in this study to detect target organisms within spiked environmental samples by digital PCR (dPCR). Primers and probes of both extracellular DNA (exDNA) spike‐ins are compatible, and hence, they can be used within multiplex assays. Similarly, the intracellular DNA (iDNA) spike‐ins can be detected simultaneously. Hydrolysis probes are either labelled with FAM or HEX together with the Black Hole Quencher‐1 (BHQ‐1). The inner modification (5) of probes labelled with FAM describes the presence of an internal quencher 500 (IQ‐500).

Target organism	Represented state	Forward primer (5′–3′)	Reverse primer (5′–3′)	Probe (5′–3′)	Amplicon length (bp)
*E. coli ∆bglA::kan*	exDNA	TTGACGAGTTCTTCTAATAAGGGGATC	TGCATCCGGTACTTCATCGAC	HEX‐AGCAGCTCCAGCCTACAAGCAACGG‐ BHQ1	138
*B. subtilis ∆licH::kan*	exDNA	CCTGCCTTTCCTCCCTCATG	CAAAGGGGAGCAAGCATATGAC	FAM‐CCTTTCTCG5CCTGCCGTTACAATCTTCAATCC‐BHQ1	100
*E. coli ∆chbF::kan*	iDNA	TCTAGAGAATAGGAACTTCGAACTGC	TCACATATCTGTCCTGTTGCTGG	FAM‐CGGATCCCC5GGAATCATAATTTCTCCCTTCAG‐BHQ1	136
*B. subtilis ∆bglA::kan*	iDNA	GAACCAGGCACTACGGTAAAAG	CGGTAGAGAGAGCACAGATACG	HEX‐CACTGCGCTTGTTCGGATTACCAGTTGG‐BHQ1	109

#### 2.4.2 Specificity Testing and Assay Optimisation

Absolute quantification of target genes was performed by dPCR using the QIAcuity One, 5plex Device (Qiagen, Hilden, Germany) in combination with the QIAcuity Probe PCR Kit (Qiagen, Hilden, Germany) and the QIAcuity Nanoplates 8.5 k (Qiagen, Hilden, Germany). The developed primer/probe sets were obtained from Microsynth AG (Balgach, Switzerland). To experimentally evaluate the specificity of the designed assays, a dilution series of each target gene was amplified in both singleplex and the concomitant multiplex dPCR assays. Reaction setup and thermocycling conditions were therefore used according to the manufacturer's recommendations. Results were analysed regarding the unspecific and/or cross‐amplification between assays. Additionally, the unspecific amplification was tested for each of the four target environmental samples containing no spike‐ins. After comparing the performance of singleplex and multiplex assays, the latter were further optimised with respect to the concentration of primers/probes as well as DNA template, annealing/elongation temperature and cycle number. Moreover, the effect of DNA fragmentation on the assay performance was evaluated by adding the restriction enzyme EcoRI‐HF (New England Biolabs, Ipswich, United States) to the prepared reaction mix. Cutting sites within the target sequences were precluded beforehand.

Finally, all multiplex dPCR assays were performed in a total volume of 12 μL, containing 1X Probe PCR Master Mix, 0.8 μM of each primer, 0.4 μM of each probe, 3 U of the restriction enzyme and 1.2 μL of DNA template. Nuclease‐free water was used to bring the volume up to 12 μL. The reactions were prepared in 96‐well plates and incubated at room temperature for 10 min to allow enzymatic fragmentation of DNA templates. Subsequently, they were transferred to the wells of the nanoplate and amplified as follows: initial denaturation at 95°C for 2 min followed by 40 cycles of denaturation at 95°C for 15 s and combined annealing/elongation at 64°C for 30 s. Prior to imaging, plates were incubated at 40°C for 5 min. Raw data were analysed using the QIAcuity Software Suite v.2.5.0.1 (Qiagen, Hilden, Germany) and thresholds were set automatically to distinguish between positive and negative partitions. All samples were analysed at least in duplicate together with negative and no‐template controls.

#### 2.4.3 Quality Control of dPCR Assays

For an in‐depth assessment of the quality of the developed multiplex dPCR assays, aspects including linear regression curves, limit of detection (LoD) and limit of quantification (LoQ) were considered. For each target organism, 5‐fold dilutions of extracted gDNA were created and used as a template for dPCR reactions. For *E. coli* , the expected copy number ranged from 0.475 to 3.71 × 10^4^ μL^−1^ and 0.44 to 3.34 × 10^4^ μL^−1^ for exDNA and iDNA spike‐ins, respectively. Similarly, copy numbers ranging from 0.327 to 2.56 × 10^4^ μL^−1^ and from 0.303 to 2.37 × 10^4^ μL^−1^ were expected for spiked exDNA and iDNA deriving from *B. subtilis* . Four independent dilution series were performed and analysed in triplicate to also account for inter‐specific assay variation. By plotting the number of target genes determined by dPCR against the expected dilution values, linear regression curves were generated. The LoD was defined as the lowest amount of target genes at which 95% of the replicates produced positive results. To consider also the accuracy and precision of each dPCR assay, the LoQ was defined as the lowest concentration at which all replicates produced positive results at a coefficient of variation (CV) < 35% (Klymus et al. 2020). For the determination of the LoQ, the five highest dilutions were considered. Both the LoD95% and LoQ were estimated in R v.4.2.3 (R Core Team 2023). While for the former a logit model was generated, the latter was modelled by an exponential function.

### 2.5 State‐Specific Recovery

The percent recovery of spiked eDNA states was calculated according to the following equation:

$$\%\text{recovery}=\frac{\text{number of recovered target genes}}{\text{number of spiked target genes}\;}\times 100$$

Thereby, the number of recovered target genes from spiked environmental samples was determined by dPCR and the number of spiked target genes corresponds to the number of cells and genome equivalents used as iDNA and exDNA spike‐ins, respectively, as the target genes occur solely once per cell/genome.

### 2.6 Statistical Analyses

Differences in the percent recovery were evaluated with regard to the concentration, the eDNA state and bacterial origin of spike‐ins as well as the spiked environmental samples. As data did not meet assumptions for parametric tests, including normality and homogeneity of variance, not even after data transformation, non‐parametric Kruskal‐Wallis tests were performed, restricting analyses to the main effects. Pairwise comparisons were carried out by post‐hoc tests according to Dunn using Benjamini‐Hochberg‐corrected p‐values. To test the effect of DNA fragment length of spiked exDNA on the percent recovery, a Welch‐ANOVA followed by t‐tests with unequal variance was applied as data were normally distributed but did not show homogeneity of variance between groups. All analyses were executed in R v.4.2.3 (R Core Team 2023) using the packages rstatix v.0.7.2 (Kassambara 2023), dplyr v.1.1.4 (Wickham et al. 2023) and reshape2 v.1.4.4 (Wickham 2007). Results were considered significant at *p* ≤ 5%. Figures were created in R using the package ggplot2 v.3.5.1 (Wickham 2016).

## 3 Results

### 3.1 Specificity Testing and Quality Evaluation of Multiplex dPCR Assays

Serial dilutions of the target genes were amplified in both singleplex and the concomitant multiplex dPCR assays to determine unspecific binding and cross‐amplification. For all assays tested, no amplification/fluorescence of DNA molecules other than the target genes was observed. Similarly, none of the negative controls including no‐template controls or no‐spike controls of the selected soil, sediment, sludge and compost samples showed amplification. Moreover, the number of target genes was comparable between singleplex and multiplex assays, indicating a similar performance (spiked exDNA: *p*_Wilcoxon/ *E.coli*_ = 0.401, *p*_Wilcoxon/ *B.subtilis*_ = 0.845; spiked iDNA: *p*_Wilcoxon/*E.coli*_ = 0.539, *p*_Wilcoxon/ *B.subtilis*_ = 0.263). The quality of the designed assays was further evaluated with regard to the LoD95%, LoQ and linear regression curves of the respective dilution series. The logit analyses revealed a LoD95% of 19 copies/reaction (95% confidence interval (CI): 12.7–53.4) and 18 copies/reaction (95% CI: 12.2–50.9) for spiked exDNA deriving from *E. coli* and *B. subtilis* , respectively. In the case of iDNA spike‐ins, for *E. coli* , 17 copies/reaction (95% CI: 12.9–30.0) and for *B. subtilis* , 13 copies/reaction (95% CI: 8.8–29.6) were determined as LoD95%. As modelled by exponential functions, the criterion for a CV lower than 35% was met at 85 copies/reaction ( *E. coli* , 95% CI: 73.9–99.1) and 71 copies/reaction ( *B. subtilis* , 95% CI: 62.0–82.5) for spiked exDNA as well as 85 copies/reaction ( *E. coli* , 95% CI: 74.6–98.6) and 63 copies/reaction ( *B. subtilis* , 95% CI: 53.9–74.4) for spiked iDNA. Linear regression curves were generated by plotting the mean number of target genes determined by dPCR against the known values of the dilution series. For concentrations above LoD95%, a high linearity (*R* ^2^ > 0.998) was achieved for all assays ( *E. coli* spiked exDNA: *R* ^2^ = 0.9998; *B. subtilis* spiked exDNA: *R* ^2^ = 0.9982; *E. coli* spiked iDNA: *R* ^2^ = 0.9994; *B. subtilis* spiked iDNA: *R* ^2^ = 0.9993) (Figure S1).

### 3.2 Recovery of Spiked eDNA States

**FIGURE 2:**
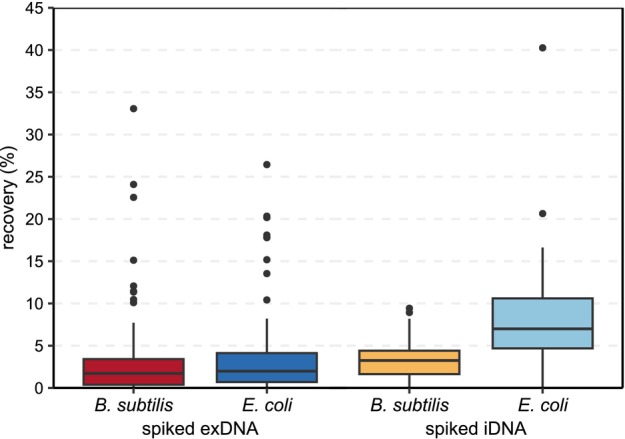
Effect of the state of environmental DNA (eDNA, i.e., intracellular DNA = iDNA and extracellular DNA = exDNA) and its bacterial origin (i.e., *B. subtilis* = gram‐positive and *E. coli* = gram‐negative) on the percent recovery of spike‐ins (*p*_Kruskal‐Wallis_ < 0.001).

The applicability of the designed spike‐and‐recovery controls was tested within soil, sediment, sludge and compost samples. All of them were chosen randomly and did not show amplification of the target genes. To assess the minimum number of added spike‐ins which is necessary to reliably track the respective target genes within environmental samples, several numbers of cells and genome equivalents, ranging from 10^5^ to 10^8^ were evaluated, respectively. Regardless of the spiked eDNA state, its bacterial origin and the environmental sample tested, the percent recovery of target genes was similar among the different concentrations (*p*_Kruskal‐Wallis_ = 0.450). However, only the concentration of 10^8^ revealed positive results, i.e., detection above LoD95% for all target genes, environmental samples and replicates tested. At this concentration, the mean percent recovery was 4.9% ± 4.34%, indicating that on average 95% of iDNA and exDNA spike‐ins were lost during DNA extraction or could not be amplified by dPCR. Similarly, no concentration‐dependent effects were determined by separate analyses of the spiked eDNA states of each model organism ( *E. coli* spiked exDNA: *p*_Kruskal‐Wallis_ = 0.610; *B. subtilis* spiked exDNA: *p*_Kruskal‐Wallis_ = 0.635; *E. coli* spiked iDNA: *p*_Kruskal‐Wallis_ = 0.647; *B. subtilis* spiked iDNA: *p*_Kruskal‐Wallis_ = 0.739). Hence, the number of spike‐ins was not considered for further analyses. Comparing the percent recovery among target genes irrespective of the environmental samples tested, significant differences were observed between both the spiked eDNA states and their bacterial origin (*p*_Kruskal‐Wallis_ < 0.001) (Figure 2). Post‐hoc tests revealed that the recovery of spiked iDNA originating from *E. coli* was highest (7.8% ± 7.01%) and differed significantly from *B. subtilis* (3.3% ± 2.37%, *p*_Dunn_ = 0.007) as well as spiked exDNA of both *E. coli* (4.8% ± 6.78%, *p*_Dunn_ = 0.004) and *B. subtilis* (4.4% ± 7.01%, *p*_Dunn_ = 0.001). In the case of spiked exDNA, however, the percent recovery of target genes was similar for both *E. coli* and *B. subtilis* (*p*_Dunn_ = 0.714). Likewise, none of them differed significantly from iDNA spike‐ins coming from *B. subtilis* (*p*_Dunn_ = 0.770 and 0.615, respectively). Obviously, separate analyses for each environmental sample changed the observed effects slightly. For example, the recovery of spiked iDNA was significantly higher for *E. coli* than for *B. subtilis* in soil (*p*_Dunn_ = 0.003) and sludge samples (*p*_Dunn_ = 0.002), while similar results were obtained for compost (*p*_Dunn_ = 0.155) and sediment samples (*p*_Kruskal‐Wallis_ = 0.965). However, the bacterial origin did not significantly influence the recovery of spiked exDNA in any of the four selected target environments (Table S3). As expected, the overall percent recovery of spiked eDNA states differed significantly between the environmental samples (*p*_Kruskal‐Wallis_ < 0.001). Independent of the target genes, the highest amounts were recovered from sludge samples (11.6% ± 8.40%), which were on average 19.3‐, 2.9‐ and 2.8‐times higher than those recovered from sediment (0.6% ± 1.54%), soil (4.0% ± 2.75%) and compost samples (4.1% ± 3.81%), respectively. Hence, post‐hoc tests revealed significant differences between all of them except for soil and compost samples (Figure S2).

**FIGURE 3:**
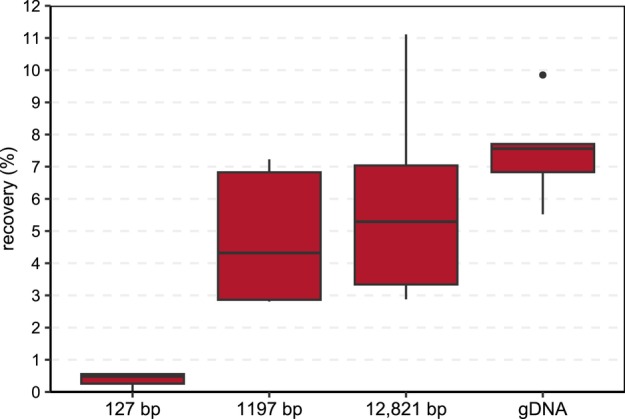
Effect of DNA fragment length on the percent recovery of spiked extracellular DNA (exDNA, *p*_Welch‐ANOVA_ < 0.001). A total of 127, 1197 and 12,821 bp fragments were generated by PCR amplification, while gDNA refers to genomic DNA, which was isolated from pure bacterial cell cultures.

To test whether the length of spiked exDNA affects its recovery, PCR products of 127, 1197 and 12,821 bp as well as gDNA were used as spike‐ins. However, in this context, solely sludge and soil samples were considered, as previous experiments showed that sediment samples yielded the least percent recovery of all target genes, and compost samples achieved similar results compared to soil samples (Figure S2). Overall, the percent recovery of spiked exDNA differed significantly with respect to its fragment length (*p*_Welch‐ANOVA_ < 0.001) (Figure 3). Pairwise comparisons showed that a significantly lower number of the spiked 127 bp fragment (0.4% ± 0.24%) was recovered compared to the 1197 bp (*p*_*t*‐test_ = 0.012) and 12,821 bp fragments (*p*_*t*‐test_ = 0.017) as well as gDNA (*p*_*t*‐test_ < 0.001). For those three spike‐ins, the mean percent recovery was similar, obtaining values of 4.8% ± 2.17%, 5.8% ± 3.16% and 7.5% ± 1.43%, respectively. Separate analyses on both environmental samples revealed similar results for sludge samples, while significant differences were observed between all spike‐ins, except for the 1197 and 12,821 bp fragments within soil samples.

## 4 Discussion

### 4.1 Requirements for Spike‐and‐Recovery Controls

The use of spike‐and‐recovery controls to account for DNA recovery efficiency is widely acknowledged in molecular microbial ecology; however, its implementation remains laborious since several requirements have to be satisfied. Besides being naturally absent in the investigated environments, spike‐ins have to behave similarly to the focal organisms (Harrison et al. 2021; Stoeckel et al. 2009). Due to the ubiquitous distribution of microorganisms, several strategies have been implemented to circumvent these issues. While some studies used exotic organisms, which naturally do not occur in the investigated environment (e.g., Kirtane et al. 2023; Stämmler et al. 2016), others combusted and/or sterilised the environmental samples prior to the addition of spike‐ins to preclude their natural abundance (e.g., Corinaldesi et al. 2005; Geraldi et al. 2020). Moreover, splitting samples and comparing results of spiked and non‐spiked samples has also been reported to indirectly estimate the recovery of spike‐ins (e.g., Deshpande and Fahrenfeld 2022; Wang et al. 2016). However, these strategies often multiply the expenses in the context of laboratory work and costs, potentially inflate the accurate quantification and alter environmental properties not reflecting the true in situ conditions anymore (Alawi et al. 2014; Geraldi et al. 2020). The methodological approach proposed in this study circumvents these drawbacks and successfully meets the requirements for reliable spike‐and‐recovery controls without altering the habitat conditions. Even though the chosen model organisms *E. coli* and *B. subtilis* are ubiquitously spread in the environment (Akinsemolu et al. 2024; Earl et al. 2008; Jang et al. 2017; van Elsas et al. 2011), the selected single‐gene deletion mutants were naturally absent in the investigated environmental samples, including soil, sediment, sludge and compost, emphasising the high specificity of the designed assays. These strains were genetically modified in the laboratory by replacing non‐essential genes with an antibiotic resistance cassette (Baba et al. 2006; Koo et al. 2017). Hence, each antibiotic resistance gene occurs solely once within each bacterial cell, allowing its precise quantification compared to for example, plasmid‐encoded genes, which can occur manifold within cells (Jahn et al. 2016; McKinney and Dungan 2020; Stoeckel et al. 2009). Moreover, the applied construct enables the design of specific primer and probe sets for each target gene, allowing the simultaneous spiking of different bacterial groups and eDNA states. In this context, Kirtane et al. (2023) showed that the percent recovery did not differ depending on whether the different eDNA states were added individually or together with other eDNA states to the environmental samples. Our proposed spike‐and‐recovery controls were implemented for dPCR but may easily be adapted to real‐time PCR (qPCR) as well. However, the major advantage of dPCR over qPCR is that it is far simpler to use for the absolute quantification of target genes as there is no need to run internal calibration curves, emphasising its robustness when comparing results across studies and laboratories (Emerson et al. 2017; Harrison et al. 2021; The dMIQE Group and Hugget 2020; Wang et al. 2023).

### 4.2 Effect of Bacterial Origin on DNA Recovery

The simultaneous tracking of both gram‐positive and gram‐negative bacteria allows more profound insights into species‐dependent DNA extraction and recovery, which was mainly obvious in the case of spiked iDNA (Figure 2). Indeed, the percent recovery of spiked iDNA originating from *E. coli* was higher than that for *B. subtilis* , highlighting the remarkable differences in the accessibility of bacterial cell walls to cell lysis. Notwithstanding, the chosen DNA extraction kit (NucleoSpin Soil Kit, Macherey‐Nagel) was previously shown to perform superiorly within different environments including soil, sludge and manure compared to other extraction kits (e.g., Desneux and Pourcher 2014; Knauth et al. 2013; Roopnarain et al. 2017; Wagner et al. 2015). In this context, Roopnarain et al. (2017) emphasised that the combined mechanical and chemical lysis, as performed by the applied DNA extraction kit, was most effective at lysing hard‐to‐lyse cells, such as gram‐positive bacteria. Even though an increase in bead‐beating may increase the percent recovery, this may concomitantly result in DNA shearing/fragmentation of lysis‐sensitive bacteria (Albertsen et al. 2015; Robe et al. 2003). Hence, a trade‐off between efficient cell lysis while maintaining DNA integrity has to be made. Indeed, eukaryotic microorganisms such as fungi are known to be even more recalcitrant to cell lysis than bacteria due to the structural differences in their cell walls conferring higher rigidity (Kollu and LaJeunesse 2021; Starke et al. 2019; Taubert et al. 2000). However, their use as spike‐and‐recovery controls remains challenging due to the lack of appropriate methods to accurately quantify filamentous, multicellular organisms prior to their use as spike‐ins, and therefore, they have to be part of further investigations.

### 4.3 Effect of eDNA State on DNA Recovery

Compared to the species‐specific differences in the recovery of spiked iDNA, the overall percent recovery was similar for both *E. coli* and *B. subtilis* in the case of spiked exDNA (Figure 2). Obviously, the structure and hence, the possible fates (e.g., persistence, biotic/abiotic degradation) in the environment are similar for DNA molecules irrespective of their gram‐negative or gram‐positive origin. For example, while eukaryotic DNA is tightly wrapped around histones, prokaryotic DNA interacts with histone‐like proteins (Torti et al. 2015). Interestingly, the percent recovery of spiked exDNA was found to be lower than that of spiked iDNA deriving from *E. coli* , even though the latter is enclosed within the cell envelope conferring additional protection. However, exDNA may also bind onto organic and inorganic surfaces within the environment, hampering their accessibility to DNA extraction. For example, mineralogy, pH and ionic strength of the environmental sample are among the driving factors influencing DNA adsorption (Carini et al. 2016; Kirtane et al. 2023; Levy‐Booth et al. 2007; Pietramellara et al. 2009; Romanowski et al. 1991). Under circumneutral conditions, both the adsorbing surfaces and the DNA molecules are negatively charged, and therefore, DNA adsorption relies on the formation of cation bridges. Hence, the concentration and charge of cations determine the persistence of DNA, with bivalent cations being more effective than monovalent cations (Levy‐Booth et al. 2007; Pietramellara et al. 2001, 2009; Romanowski et al. 1991; Saeki et al. 2010; Torti et al. 2015). Romanowski et al. (1991) reported that under these conditions, the adsorption of DNA molecules occurs rapidly, as more than 85% of added DNA was adsorbed to chemically pure sand in the presence of MgCl_2_ in as little as 1 min. However, natural environments are likely to be more complex and heterogeneous than these clearly defined and well‐characterised sorbents (Ogram et al. 1994; Pietramellara et al. 2009). That is why one parameter alone can hardly predict the adsorption behaviour of DNA molecules in complex environments. Instead, it is a myriad of different, interacting parameters. Indeed, the percent recovery of spiked eDNA states differed also between the environmental samples tested in this study. However, deciphering underlying patterns for each of them would be beyond its scope, as the main aim was to establish spike‐and‐recovery controls accounting for both the different eDNA states and bacterial origins within natural environments. In this context, however, Geraldi et al. (2020) proposed testing different concentrations of spike‐ins, mainly in the case of exDNA, as too high concentrations perhaps prevent DNA adsorption, exceeding the limits of binding capacity. Among the different spike‐in concentrations tested (10^5^, 10^6^, 10^7^, 10^8^ cells and genome equivalents, respectively), no differences in percent recovery were observed, assuming that even the highest amount of 10^8^ genome equivalents did not cause saturation. In fact, this amount was actually needed to reach detection above LoD95% within all environmental samples tested. Moreover, it is suggested that DNA adsorption and thus, the percent recovery depend on the properties of the DNA molecules themselves, such as conformation, purity and length (Ogram et al. 1994; Pietramellara et al. 1997, 2001, 2007, 2009). Exemplarily, we tested the effect of fragment length on the recovery of spiked exDNA within selected environmental samples. Overall, no significant differences were observed between spiked PCR products of 1197 bp, 12,821 bp and gDNA, while the percent recovery of the 127 bp fragment was significantly reduced (Figure 3). This is in agreement with Alawi et al. (2014), who found that the recovery efficiency of DNA fragments spiked into phosphate buffer increased with increasing fragment length. Similarly, it was reported that DNA adsorption increased with decreasing fragment size, as smaller fragments are able to enter small pores and microaggregates within the environment, receiving higher protection against enzymatic degradation (Ogram et al. 1994; Pietramellara et al. 1997, 2001). Likewise, this may reduce their accessibility to DNA extraction, as shown in the present study.

## 5 Conclusion and Future Research

Although the proposed methodological approach was specifically designed for bacteria and not yet validated within further environmental samples (e.g., aquatic environments), it provides a valuable step toward the simplified and reliable application of spike‐and‐recovery controls for DNA extraction which do not only account for the different eDNA states, but also for the bacterial accessibility to cell lysis in the case of spiked iDNA. To the best of our knowledge, this is the first study considering all these aspects within a single experiment. Hence, future studies may benefit from these easy‐to‐implement spike‐in controls to compare the performance of different DNA extraction methods/kits on environmental samples and to determine the effects of inhibitors while assessing species‐specific differences and related consequences on subsequent downstream analyses. However, irrespective of the eDNA state and their bacterial origin, the overall percent recovery of spike‐ins obtained by the extraction of the total eDNA was rather low. Even though the causes are probably manifold, a promising strategy to enhance the DNA yields is the separation of the different eDNA states. As shown previously, higher amounts of extractable DNA were achieved in this way compared to the direct extraction and analysis of the total eDNA (Alawi et al. 2014; Ascher et al. 2009; Ceccherini et al. 2009; McKinney and Dungan 2020; Nagler, Podmirseg, et al. 2018). Indeed, a multitude of methodological approaches for the separation of exDNA and iDNA from environmental samples has been published (e.g., Alawi et al. 2014; Ascher et al. 2009; Lever et al. 2015; Nagler, Podmirseg, et al. 2018; Ogram et al. 1987). Even though many of these protocols are conceptually similar, their validation is critical and hardly acknowledged due to the lack of appropriate experimental setups. However, the herein proposed spike‐and‐recovery approach could circumvent many of these sample‐specific and species‐specific problems and meet the requirements that are needed for the reliable and accurate validation of such protocols, advancing our understanding of the structure and dynamics of microbial communities and thus the correct interpretation of eDNA‐based studies. Indeed, research on the separation of the different eDNA states and the concomitant validation and optimisation of extraction protocols using the proposed spike‐and‐recovery controls is currently in process and therefore will deserve further attention in future studies.

## Author Contributions

**Julia Zöhrer:** conceptualization, methodology, data curation, visualization, writing – original draft, investigation, formal analysis. **Judith Ascher‐Jenull:** conceptualization, supervision, writing – review and editing. **Andreas O. Wagner:** conceptualization, funding acquisition, writing – review and editing, supervision, resources, project administration.

## Conflicts of Interest

The authors declare no conflicts of interest.

## Supporting information


**Data S1.** Supporting Information.

## Data Availability

The data presented in this study are available on figshare (10.6084/m9.figshare.28563458).
