# Supplementary material for: Tracking Different States of Spiked Environmental DNA Using Multiplex Digital PCR Assays
Source: Environ Microbiol. 2025 Mar 28;27(4):e70086. doi: 10.1111/1462-2920.70086 (PMC11950903; doi:10.1111/1462-2920.70086)
Supplement: Supplementary file 1 — Data S1. Supporting Information. [file EMI-27-e70086-s001.docx]

**Tracking different states of spiked environmental DNA using multiplex digital PCR assays**

Julia Zöhrer^1^*, Judith Ascher-Jenull^2^, Andreas O. Wagner^1^

^1^Department of Microbiology, Universität Innsbruck, Innsbruck, Austria

^2^Department of Experimental Architecture, Integrative Design Extremes, Universität Innsbruck, Innsbruck, Austria

*Corresponding author: [julia.zoehrer@uibk.ac.at](mailto:julia.zoehrer@uibk.ac.at)

**Supplementary Material**

Supplementary Tables (Table S1, Table S2, Table S3)

Supplementary Figures (Figure S1, Figure S2)

**Supplementary Tables**

**Table S1.** Primer sequences for the generation of differentially sized PCR products. Genomic DNA isolated from B. subtilis ∆licH::kan was used as DNA template.

| **Forward primer (5’ – 3’)** | **Reverse primer (5’ – 3’)** | **Amplicon length (bp)** |
| --- | --- | --- |
| TGCTATACGAACGGTATCCTGC | AATCACCGAGCAAAGGGGAG | 127 |
|  | CGGCTGATTGTTGAAGCGAC | 1197 |
|  | CGCGGTCTATTTGCATGGTG | 12821 |

**Table S2.** Primer sequences for the amplification and sequencing of genomic regions targeted by dPCR assays. PCR amplicons including the kanamycin resistance cassette and approximately 1 kb of the 5’ and 3’ flanking regions were generated and sequenced.

| **Target organism** | **Forward primer (5‘ – 3‘)** | **Reverse primer (5‘ – 3‘)** | **Amplicon length (bp)** | **Sequencing primers (5‘ – 3‘)** |
| --- | --- | --- | --- | --- |
| *E. coli ∆bglA::kan* | CATAAATTACCACCAGCGACAG | GGAAAAGCGTGCCAACATTG | 3199 | CATAAATTACCACCAGCGACAG |
|  |  |  |  | GGAAAAGCGTGCCAACATTG |
|  |  |  |  | GTCAGCGTATCCAGCGTTAC |
|  |  |  |  | TTTGTCAAGACCGACCTGTC |
| *B. subtilis ∆licH::kan* | GTTCGGAAGAGTGTTTACAGACC | ATTCTGGGAGGAAGCCTTGG | 3172 | GTTCGGAAGAGTGTTTACAGACC |
|  |  |  |  | ATTCTGGGAGGAAGCCTTGG |
|  |  |  |  | CAATTCAGTCATCGCCTTGC |
|  |  |  |  | GATGGAGTGAAAGAGCCTGATG |
| *E. coli ∆chbF::kan* | CGCCGCCTCAAATGATTTAC | CCTGCCATTGTTGCCTTATTG | 2961 | CGCCGCCTCAAATGATTTAC |
|  |  |  |  | CCTGCCATTGTTGCCTTATTG |
|  |  |  |  | AGATCCATTTTCCCAGCACAC |
|  |  |  |  | CAGGAGCAAGGTGAGATGAC |
| *B. subtilis ∆bglA::kan* | GTAAAGGCATCCGAACTGGTG | GATTCTTCGTAGCAAAGGGTCTG | 3039 | GTAAAGGCATCCGAACTGGTG |
|  |  |  |  | GATTCTTCGTAGCAAAGGGTCTG |
|  |  |  |  | TGGTTAGAAGGAACGGTTGAAC |
|  |  |  |  | GATGGAGTGAAAGAGCCTGATG |

**Table S3.** Overview of the percent recovery of extracellular and intracellular spike-ins as a function of the selected environmental samples. Values are means with standard deviations. For each environment, different letters (in brackets) indicate significant differences (p < 0.05; Kruskal-Wallis tests followed by post-hoc tests according to Dunn) between spike-ins.

|  | **spiked exDNA (% recovery)** | | **spiked iDNA (% recovery)** | |
| --- | --- | --- | --- | --- |
|  | ***B. subtilis*** | ***E. coli*** | ***B. subtilis*** | ***E. coli*** |
| **compost** | 0.9 ± 0.88 (a) | 1.3 ± 1.03 (a) | 5.4 ± 1.87 (b) | 8.7 ± 3.40 (b) |
| **sediment** | 0.5 ± 1.14 (a) | 0.8 ± 1.64 (a) | 0.3 ± 0.86 (a) | 0.8 ± 2.27 (a) |
| **sludge** | 13.8 ± 8.72 (a) | 14.8 ± 6.84 (a) | 4.2 ± 1.98 (b) | 13.8 ± 9.66 (a) |
| **soil** | 2.3 ± 0.65 (a) | 2.5 ± 0.89 (a) | 3.1 ± 0.57 (a) | 8.1 ± 2.50 (b) |

**Supplementary Figures**

**Figure S1.** Quality control of designed multiplex dPCR assays for exDNA spike-ins (A-F) as well as spiked iDNA (G-L) originating from *B. subtilis* (A-C, G-I) and *E. coli* (D-F, J-L). The LoD95% was determined by logit analyses (A, D, G, J). The y-axis shows the fraction of positive results obtained at a given concentration of the dilution series, indicated on the x-axis. Dashed lines represent the 95% confidence interval and dotted lines refer to the number of target copies with an estimated probability of detection of 95%. The LoQ was modelled by exponential functions (B, E, H, K). The y-axis shows the CV (standard deviation/mean, n = 12) obtained at a given number of target copies. Dashed lines represent the 95% confidence interval and dotted lines refer to the number of target copies where the CV is 35%. Linear regression curves were created by plotting the number of target copies measured by dPCR against the anticipated values of the dilution series (C, F, I, L). Data are shown as means and standard deviations (n = 12) for concentrations above the LoD95%.

**Figure S2.** Effect of the environmental sample on the percent recovery of spike-ins (p_Kruskal-Wallis_ < 0.001).
